# In Situ Studies on the Influence of Surface Symmetry on the Growth of MoSe_2_ Monolayer on Sapphire Using Reflectance Anisotropy Spectroscopy and Differential Reflectance Spectroscopy

**DOI:** 10.3390/nano14171457

**Published:** 2024-09-07

**Authors:** Yufeng Huang, Mengjiao Li, Zhixin Hu, Chunguang Hu, Wanfu Shen, Yanning Li, Lidong Sun

**Affiliations:** 1 State Key Laboratory of Precision Measurement Technology and Instruments, School of Precision Instrument and Opto-Electronics Engineering, Tianjin University, Tianjin 300072, China; 2 Tianjin Key Laboratory of Low Dimensional Materials Physics and Preparing Technology, Department of Physics, Center for Joint Quantum Studies, Tianjin University, Tianjin 300350, China; 3 Institute of Experimental Physics, Johannes Kepler University Linz, A-4040 Linz, Austria

**Keywords:** reflectance anisotropy spectroscopy, differential reflectance spectroscopy, MoSe_2_, real-time monitoring, molecular beam epitaxy

## Abstract

The surface symmetry of the substrate plays an important role in the epitaxial high-quality growth of 2D materials; however, in-depth and in situ studies on these materials during growth are still limited due to the lack of effective in situ monitoring approaches. In this work, taking the growth of MoSe_2_ as an example, the distinct growth processes on Al_2_O_3_ ($11\bar{2}0$) and Al_2_O_3_ (0001) are revealed by parallel monitoring using in situ reflectance anisotropy spectroscopy (RAS) and differential reflectance spectroscopy (DRS), respectively, highlighting the dominant role of the surface symmetry. In our previous study, we found that the RAS signal of MoSe_2_ grown on Al_2_O_3_ ($11\bar{2}0$) initially increased and decreased ultimately to the magnitude of bare Al_2_O_3_ ($11\bar{2}0$) when the first layer of MoSe_2_ was fully merged, which is herein verified by the complementary DRS measurement that is directly related to the film coverage. Consequently, the changing rate of reflectance anisotropy (RA) intensity at 2.5 eV is well matched with the dynamic changes in differential reflectance (DR) intensity. Moreover, the surface-dominated uniform orientation of MoSe_2_ islands at various stages determined by RAS was further investigated by low-energy electron diffraction (LEED) and atomic force microscopy (AFM). By contrast, the RAS signal of MoSe_2_ grown on Al_2_O_3_ (0001) remains at zero during the whole growth, implying that the discontinuous MoSe_2_ islands have no preferential orientations. This work demonstrates that the combination of in situ RAS and DRS can provide valuable insights into the growth of unidirectional aligned islands and help optimize the fabrication process for single-crystal transition metal dichalcogenide (TMDC) monolayers.

## 1. Introduction

Two-dimensional (2D) transition metal dichalcogenides (TMDCs) have attracted a tremendous amount of attention due to their atomically thin thickness, excellent electronic and optoelectronic properties [1,2], and unique band structures [3,4]. Aside from having similar carrier high mobility to MoS_2_, 2D MoSe_2_ exhibits higher electrical conductivity, stronger spin–orbit [5], and narrower bandgap, giving it more potential for the application of energy storage [6], catalysis [7], and optoelectronics [8]. Numerous studies have focused on the synthesis of monolayer single-crystalline MoSe_2_ using chemical vapor deposition (CVD) and molecular beam epitaxy (MBE) [9,10,11,12,13,14]. Sapphire is commonly used as the substrate for the aligned growth of TMDC layers due to the small lattice mismatches between sapphire and TMDC materials and its atomically smooth step. Step edges of the substrate can serve as preferential nucleation sites, which make MoSe_2_ domains nucleate along the step edge direction on c-plane sapphire during CVD growth [15,16,17]. The MoSe_2_ layers grown on c-plane sapphire via MBE show a homogeneous and crystalline structure, whereas single MoSe_2_ domains exhibit free rotation angles [18,19]. In addition, both density functional theory (DFT) calculations [20] and experimental research, such as the fabrications of single-crystal WS_2_ monolayer [21], highly orientated MoS_2_ [22] and MoSe_2_ [23] layers on a-plane sapphire, have shown that using a low-symmetry substrate is an effective strategy in the oriented growth of TMDC monolayer.

Despite abundant achievements in the single oriented growth of 2D TMDC materials, a precise knowledge of the growth mechanisms and the control over the growth of the 2D TMDC films remains challenging. To date, both structural and optical characterization techniques have been developed to achieve in situ monitoring during the growth of 2D TMDCs. Low-energy electron microscopy (LEEM) has been applied to investigate the growth kinetics of 2D materials such as graphene and *h*-BN, including the observation of morphology or orientation evolution, and the quantitative analysis of growth rates [24,25,26]. However, LEEM requires the sample to be conducting, which largely hinders its usage on insulating substrate, such as sapphire and silicon substate. Spectroscopic techniques like spectroscopic ellipsometry (SE), differential reflectance spectroscopy (DRS), and reflectance anisotropy spectroscopy (RAS) have demonstrated significant advantages in monitoring the growth of 2D materials due to their non-contact, non-destructive, high sensitivity and ease of integration. SE has been applied to in situ study the CVD growth of large-area graphene [27] and ex situ characterizations of 2D TMDC materials [28]. Differential reflectance spectroscopy (DRS) measures the normalized reflectance contrast between the bare substrate and the deposited thin film on the substrate afterward [29]. Recently, DRS has been successfully employed to in situ study the growth of organic [30,31] and semiconductor thin films [32]. In 2017, Wei et al. used DRS to monitor the MBE growth of MoSe_2_ thin films [33]. In addition to revealing the layer-by-layer growth mode of the MoSe_2_ film, the spectrum evolution of the oscillations allows precise control over film thickness during growth [34]. In 2019, López-Posadas et al. further developed differential transmittance spectroscopy (DTS) and utilized it for the first time to monitor the CVD growth of MoS_2_ on transparent substrate [35]. Wang et al. also established an in situ DRS setup to study the CVD growth of MoS_2_ on a SiO_2_/Si substrate [36]. While DRS delivers the normalized reflectance changes during growth, RAS measures the normalized difference of the linearly polarized reflectance along two orthogonal directions [37]. These two methods are complemented. The RAS has been widely used for in situ and ex situ studies of surfaces or interfaces, including complex organic films [38,39], semiconductors [40,41], and metal thin films [42], and solid–liquid interfaces [43]. Moreover, RAS has been treated as an effective tool to investigate the anisotropic optical properties of low-symmetrical 2D materials and also the weak interactions between the 2D materials and the underlying substrates [44,45,46,47]. The combination of RAS and DRS has been utilized and discussed for a wide range of organic and inorganic semiconductor materials [48,49], and thus, in situ and real-time studies merit exploration during the growth of 2D materials.

In this work, the MBE growth of MoSe_2_ thin films on Al_2_O_3_ ($11\bar{2}0$) and Al_2_O_3_ (0001) is investigated, both in situ and in a real-time manner through combined RAS and DRS. Beyond our previous work, this simultaneous monitoring demonstrates that the evolution of the reflectance anisotropy (RA) intensity at 2.5 eV matches well with that of the changing rate of differential reflectance (DR) intensity of MoSe_2_ grown on Al_2_O_3_ ($11\bar{2}0$), revealing the correlation between anisotropic optical responses and the MoSe_2_ film thickness. Ex situ atomic force microscopy (AFM) demonstrates the morphological evolution of MoSe_2_ films on Al_2_O_3_ ($11\bar{2}0$) at different stages. Furthermore, in situ low-energy electron diffraction (LEED) patterns obtained from MoSe_2_ films grown on Al_2_O_3_ ($11\bar{2}0$) and Al_2_O_3_ (0001) illustrate the benefits of low-symmetry substrates in guiding the domain orientation. This study highlights the advantage of combined measurements of RAS and DRS in providing valuable insight into the mechanism of TMDC film growth, as well as the benefits of using low-symmetry substrates in guiding highly orientated TMDC layers.

## 2. Experimental Section

The molecular beam epitaxy growth of MoSe_2_ film was performed in a home-built ultra-high vacuum (UHV) system with a base pressure of 1.0 × 10^−9^ Torr. The fluxes of Se and Mo were emitted from a Knudsen cell and e-beam evaporator, with a deposition ratio of about 20:1. Single-crystal Al_2_O_3_ ($11\bar{2}0$) and Al_2_O_3_ (0001) substrates were purchased from Hefei Kejing Material Technology Corp (Hefei, China). The cleaned Al_2_O_3_ ($11\bar{2}0$) or Al_2_O_3_ (0001) substrates were baked at 1000 °C for 1.5 h to yield atomic-level steps. After being transferred into the chamber, the substrates were degassed at 700 °C for 20 min. The substrate was maintained at 530 °C during the deposition. The RAS spectrometer [50] was attached to the UHV chamber via a strain-free optical window to monitor the evolutions of optical anisotropy and optical reflectivity during the co-deposition of Mo and Se. The RA signal as a function of the deposition *t* is defined as the normalized reflectance difference at a quasi-normal incidence of linearly polarized light along two orthogonal directions within the surface plane:

(1) $$\frac{∆R_{xy}\left(t\right)}{R\left(t\right)}=\frac{R_{x}\left(t\right)-R_{y}\left(t\right)}{{\{R}_{x}\left(t\right)+R_{y}\left(t\right)\}/2},$$

where *x* and *y* denote the [0001] and [$1\bar{1}00$] axes of Al_2_O_3_ ($11\bar{2}0$) substrate, respectively. Simultaneously, the differential reflectance spectra can also be calculated using the following equation:

(2) $$\frac{∆R(t)}{R(0)}=\frac{R(t)-R(0)}{R(0)},$$

where *R*(0) and *R*(*t*) denote the total reflectance of the bare sapphire substrate surface and the one after a deposition time of *t*. Thus, the obtained DR signal $\frac{∆R}{R}$ represents the variation of the optical reflectivity with respect to the bare substrate surface as a function of the deposition time *t*. The acquisition time to collect the single RA and DR spectrum was 54 s.

The as-grown samples were characterized by LEED, AFM, Raman, and X-ray photoemission spectroscopy (XPS). In situ LEED characterizations were conducted at room temperature using Specs ErLEED 100 optics (Specs GmbH, Berlin, Germany). AFM measurements were performed using Bruker MultiMode (Bruker, Billerica, MA, USA) in a tapping mode at ambient conditions using Au-coated cantilevers with a force constant of ~2 N m^−1^. Raman spectra were performed using a Renishaw inVia reflex system (Renishaw, Dundee, IL, USA) with a 100× microscope objective lens and a 532 nm laser. XPS experiments were conducted to analyze the element composition of MoSe_2_ films using a PHI 5000 Versaprobe X-ray Al Kα (1486.6 eV) source (ULVAC-PHI, Kanagawa, Japan).

DFT calculations were carried out using the Vienna ab initio simulation package (VASP) [51]. The optB86b function was used to compute the electron exchange and correlation interaction [52]. The dispersion force was corrected with a vdW-DF scheme [53]. The wave function of electrons was described by a plane wave basis with the projector-augmented wave (PAW) method. The energy cutoff for the basis was set to 400 eV.

## 3. Results and Discussion

**Figure 1:**
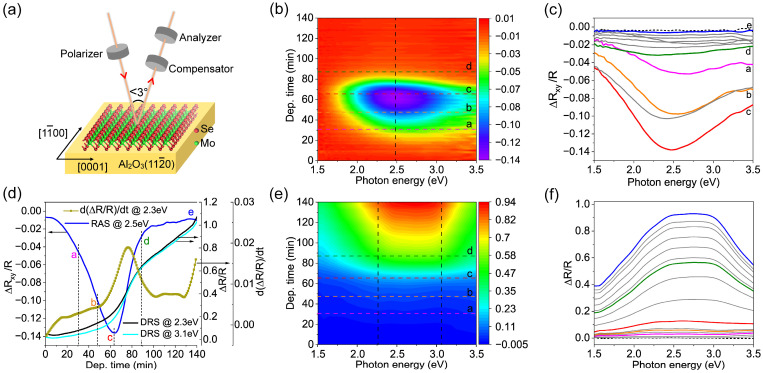
MoSe_2_ thin film growth on Al_2_O_3_ ($11\bar{2}0$) at 530 °C monitored using optical reflection measurement is schematically presented in (**a**). The setup allows the simultaneous determination of the RA and DR spectrum in real time during the growth. (**b**) Two-dimensional contour map of the RA signal over photon energy (the horizontal axis) and deposition time (the vertical axis). The RA spectra recorded at selected deposition times of *t_a_*~31 min, *t_b_*~47 min, *t_c_*~63 min, *t_d_*~87 min, and *t_e_*~140 min (indicated by the horizontal dashed lines in (**b**)), are presented in (**c**), whereas the black dotted line represents the initial RA spectrum of bare Al_2_O_3_ ($11\bar{2}0$) substrate. The evolution of the RA intensity at 2.5 eV (along the vertical dashed line in (**b**)) is plotted in (**d**) as a function of deposition time (solid blue line). In a similar fashion, the corresponding DR spectra are exhibited, namely, 2D contour map for an overview in (**e**), spectra recorded at *t_a_*, *t_b_*, *t_c_*, *t_d_*, and *t_e_* in (**f**), and the variation of the DR signals as a function of the deposition time at 2.3 eV and 3.1 eV in (**d**). The first derivative curve of the change in RA intensity at 2.5 eV is also shown in (**d**).

The molecular beam epitaxy (MBE) growth of MoSe_2_ on Al_2_O_3_ ($11\bar{2}0$) substrate was monitored by parallel measurements of RAS and DRS, and the results are shown in Figure 1. Figure 1a illustrates the setup of simultaneous monitoring using the RAS and DRS method. Figure 1b shows the dynamic evolution of the RA signal during the growth of MoSe_2_ adlayer, revealing a nonmonotonic variation. A broad RA peak around 2.5 eV grows in the first 63 min of deposition. However, further deposition leads to the intensity decay of this RA peak, which eventually almost vanishes after 90 min, a moment we associate with the completion of the first continuous MoSe_2_ monolayer [23], which was also verified by the LEED and AFM results. Indeed, Figure 1c,d show this behavior more clearly in terms of spectral line shape and time evolution, respectively. Therefore, based on the evolution of RA intensity at 2.5 eV as a function of deposition time (the blue line in Figure 1d), the deposition of MoSe_2_ can be divided into three parts: the negative growth in the first stage (*t_a_* − *t_c_*), the subsequent rapid decline (*t_c_* − *t_d_*) in the second stage, and the saturation in the final stage (*t_d_* − *t_e_*). In contrast to the RAS, the simultaneously recorded DRS (Figure 1e,f) shows a monotonic increase throughout the entire deposition. However, the deposition can still be divided into three sections based on the slope of the increase. Most importantly, the three sections defined by both methods show a clear coincidence, which is more obvious from the derivative curve of the RA signal in Figure 1d.

**Figure 2:**
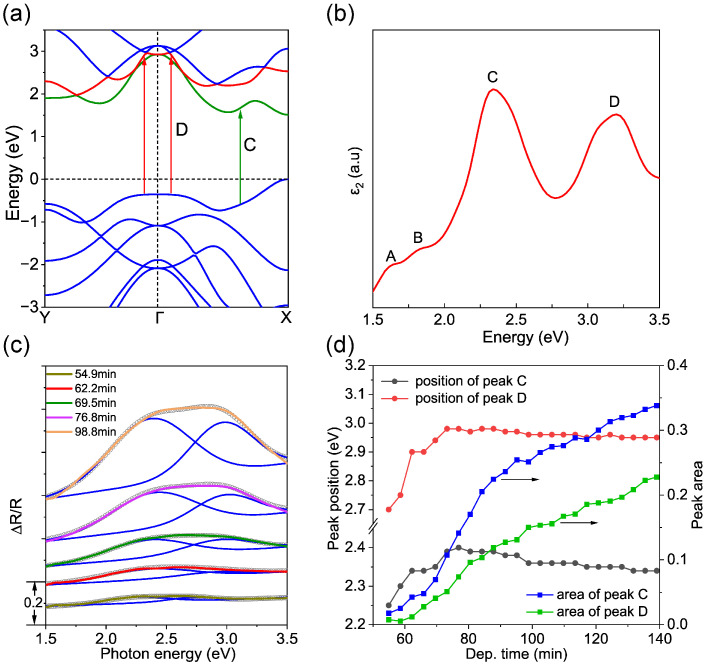
(**a**) The band structure of monolayer MoSe_2_ calculated by DFT. The arrows indicate the transition in C and D from the valance band to the conduction band. (**b**) The imaginary part of the calculated dielectric function for monolayer MoSe_2_. The main features are labeled as A to D. (**c**) Representative DR spectra recorded from the growth of MoSe_2_ on Al_2_O_3_ ($11\bar{2}0$) surface. The dark circles and blue lines represent the Lorentz fit of DR spectra recorded at different times. (**d**) The evolutions of center energy and integral area of peaks C and D as a function of deposition time.

It is worth noting that the Al_2_O_3_ ($11\bar{2}0$) substrate is transparent in the visible range. The DRS spectra in Figure 1 display a direct view of the absorptions of the MoSe_2_ adlayer [54]. Consequently, Figure 1e,f reveal the spectroscopic evolution of the optical absorption of the MoSe_2_ layer during growth. Indeed, the DR spectra presented in Figure 1f are in line with the ones reported in the literature, which show a pronounced broad peak centered around 2.7 eV [55]. To have a deep understanding of the absorption spectrum observed, DFT calculations were carried out based on the grown 2H-phase MoSe_2_ monolayer. The calculated electronic band structure around the Γ point of monolayer MoSe_2_ is presented in Figure 2a, whereas the corresponding imaginary part of the dielectric function *ε*_2_ is plotted in Figure 2b. By comparing Figure 2b with Figure 1f, it is clear that the absorption spectra of the MoSe_2_ adlayer during growth are dominated by its characteristic peaks C and D. The peaks C and D correspond to the electronic transitions located close to X and around the Γ points in the K space, which are indicated by green and red arrows, respectively. Indeed, the DR spectra can be fitted by the superposition of two Lorentz oscillators with their peak positions located close to 2.4 eV and 3 eV (see Figure 2c). Furthermore, we found that the position of both C and D peaks shift with deposition time as demonstrated in Figure 2d, indicating the slight variation of the electronic/optical properties of the MoSe_2_ adlayer as a function of coverage ratios. In agreement with the evolution of DR intensity shown in Figure 1d, the areas of peaks C and D likewise show a nonlinear increase with time. It can be also recognized from Figure 1d that the increase in the RA intensity at the initial stage of the deposition is rather slow, and the characteristic spectral line shape becomes well distinguished only after *t_b_*, which is about *t* = 50 min. The nonlinear dependence of the DR intensity and the shift of the peak position demonstrate the variation of the optical property of the actual growing MoSe_2_ adlayer. We tentatively attribute this behavior to the influence of the MoSe_2_ adlayer morphologies and lattice structure. Furthermore, it has been demonstrated that the excitonic features A and B of monolayer MoSe_2_ broadened and redshifted with the increase in temperature [56,57], which can explain the absence of peaks A and B in the DRS spectra recorded at an elevated temperature of 530 °C (Figure 1f).

**Figure 3:**
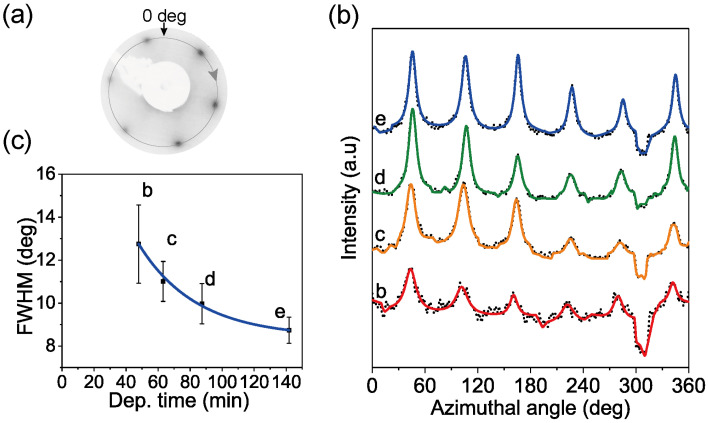
LEED results of as-grown MoSe_2_ sample. (**a**) LEED pattern of MoSe_2_ thin film (after the deposition time of *t_e_*) using an electron beam energy of 135 eV. (**b**) The line profiles (dotted lines) taken across the six first-order diffraction spots on LEED patterns (indicated by the arrowed gray circle in (**a**)) obtained after various deposition times of *t_b_*, *t_c_*, *t_d_*, and *t_e_*, respectively. The lines are normalized and offset for clarity. (**c**) The variation of the FWHM obtained by fitting the experimental profiles (solid lines) in (**b**) as a function of the deposition time.

In order to verify our above inferences, firstly, the lattice structure of the MoSe_2_ adlayer was investigated using LEED, in situ, directly after the deposition time of *t_a_*, *t_b_*, *t_c_*, *t_d_*, and *t_e_*. The characteristic hexagonal lattice structure of MoSe_2_ appears already after a deposition time of *t_b_* (see Figure 3), indicating the formation of singly oriented MoSe_2_ monolayer islands. On the other hand, the FWHM (the full width at half maximum) decreases with deposition time, as shown in Figure 3c. This result indicates that as the MoS_2_ adlayer grows, the domain size increases and the crystalline quality improves.

**Figure 4:**
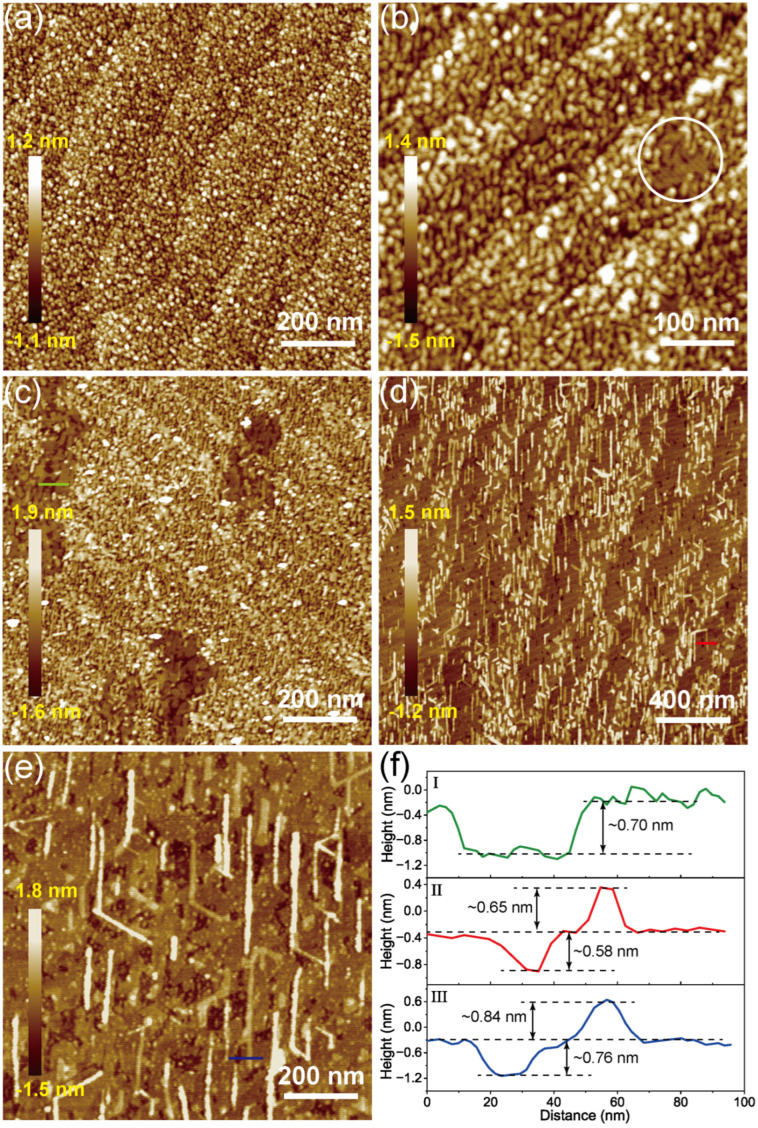
AFM images of MoSe_2_ films obtained after deposition times from *t_a_* to *t_e_* are displayed in (**a**–**e**), respectively. The white circle in (**b**) represents the coalescence of islands. (**f**) Height profiles for the green line in (**c**), the red line in (**d**), and the blue line in (**e**).

Concerning morphology, the topographic investigations of the MoSe_2_ adlayers obtained after deposition times from *t_a_* to *t_e_* were subsequently performed using AFM. The obtained adlayer after a deposition time of *t_a_* constitutes randomly distributed small islands (Figure 4a), indicating homogeneous nucleation. It can be seen that the coalescence of islands starts after *t_b_* (indicated by the white circle in Figure 4b); subsequently, the areas covered by MoSe_2_ monolayer grow with deposition time (Figure 4c) and finally complete around *t_d_* (Figure 4d). Further deposition leads to the formation of the second MoSe_2_ monolayer (Figure 4e). This observation is consistent with the XPS results, which show that the 2H-MoSe_2_ component appears at stage b (Figure S1). As seen in Figure 4c,d, the surface fraction of the monolayer covered areas gradually increases and merges into a continuous film. Here, the stages from *t_a_* to *t_b_* and *t_b_* to *t_d_* correspond to the growth and merging of MoSe_2_ monolayer islands, respectively, which satisfactorily explains the slow versus fast growth variation of the DR signal in Figure 1d. The green and red line profiles plotted in Figure 4f indicate that the depth of the gap formed during the coalescence of islands and the height of the second layer island is ~0.7 nm and ~0.65 nm, respectively. These resolved morphologies based on AFM measurements are fully in line with lattice evolutions determined by LEED. The LEED and AFM measurements thus allow us to correlate the observed evolution of the optical absorptions presented in Figure 1 with the development of the lattice and morphology of the MoSe_2_ adlayer. Indeed, the well-defined optical absorption of MoSe_2_ begins at *t_b_*, which coincides with the appearance of the monolayer islands of MoSe_2_ and the characteristic LEED pattern. Subsequently, the DR signal increases at an accelerated rate, as a result of coalescence and growth until the completion of the first monolayer. On the other hand, the DR signal increases almost linearly during the growth of the second layer of MoSe_2_, indicating a rather heterogeneous nucleation mode.

Based on the understanding so far achieved, we now consider comparing the DRS and RAS results. In contrast to the DRS, the RA signal exhibits a fast increase with a negative sign within the first ~2/3 of monolayer growth, forming a broad but distinctive negative peak around 2.5 eV. However, with continuous growth, this peak decreases rapidly in amplitude and finally saturates to a very small value. The strong optical anisotropy observed at the early stage of growth is associated with the strain accumulated in the nucleus induced by the interaction with the Al_2_O_3_ ($11\bar{2}0$) surface, which exhibits a two-fold rotation symmetry [23]. The current comprehensive information supplied by RAS and DRS supports this conclusion. This result demonstrates unambiguously the advantage of a substrate for growing highly oriented 2D TMDs. The two-fold symmetry of Al_2_O_3_ ($11\bar{2}0$) favors the formation of uniaxial nuclei at the initial stage, providing an alignment anchor for subsequent growth. On the other hand, it interacts very weakly with the 2D layer and is essentially negligible in the later stages of growth once coalescence begins, thus allowing the thermodynamic properties of the 2D layer itself to dominate the growth process, i.e., the self-assembly process.

**Figure 5:**
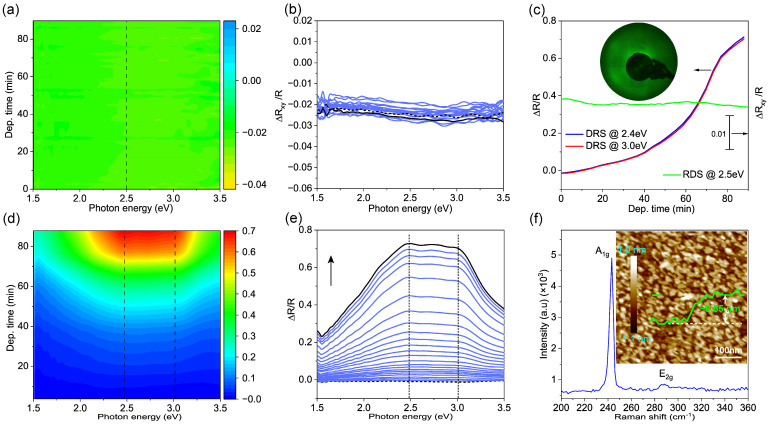
Real-time monitoring for the MBE growth of MoSe_2_ layer on Al_2_O_3_ (0001) substrate at 530 °C. (**a**) Two-dimensional contour map of the RA signal over photon energy (the horizontal axis) and deposition time (the vertical axis). (**b**) RA spectra recorded during the growth of MoSe_2_ layer on the bare Al_2_O_3_ (0001) surface (marked by black dot line) from the beginning to the end (marked by black solid line) of the growth process. The time interval is about 5 min. The evolution of the RA intensity at 2.5 eV (along the vertical dashed line in (**a**)) as a function of the deposition time is plotted in (**c**) (solid green line). The inset shows the LEED pattern of MoSe_2_ measured at an electron energy of 135 eV. The corresponding 2D contour map of DRS, the DR spectra over photon energy with about 5 min interval, and the evolution of DR intensity at 2.4 eV and 3.0 eV are shown in (**c**–**e**), respectively. (**f**) The corresponding Raman spectrum and the AFM image. The green curve represents the height profile across the green line.

To verify this conclusion, we performed reference growth on the Al_2_O_3_ (0001) surface under the same conditions, and the results are shown in Figure 5. Although the DRS also increased monotonically with deposition time (Figure 5c–e), no optical anisotropy could be detected during growth (Figure 5a,b). This can be expected from the symmetry of the Al_2_O_3_ (0001) surface. Figure 5f shows the Raman characterization of MoSe_2_ grown on Al_2_O_3_ (0001). The characteristic peaks located at 281.9 cm^−1^ and 287.1 cm^−1^ are attributed to the out-of-plane vibrational mode A_1g_ and in-plane mode E_2g_ of the MoSe_2_ layer, respectively. No peak was observed around 350 cm^−1^, indicating that the as-grown MoSe_2_ on Al_2_O_3_ (0001) surface is monolayer [58,59]. The ex situ AFM measurement is shown in Figure 5f. The profile along the green line shows that the MoSe_2_ layer is monolayer with a thickness of ~0.65 nm. Furthermore, few second-layer islands can be observed. The AFM image demonstrates that the as-grown MoSe_2_ layer has a smooth surface consisting of discontinuous monolayer islands. Thus, both Raman and AFM characterizations confirm the formation of monolayer MoSe_2_ on Al_2_O_3_ (0001). Moreover, the in situ LEED (the inset in Figure 5c) shows a diffuse ring diffraction pattern, verifying the growth of rather small grains that are not preferentially aligned on the Al_2_O_3_ (0001) surface.

## 4. Conclusions

In conclusion, the MBE growth of MoSe_2_ films on sapphire substrates terminated by two different surfaces, namely, Al_2_O_3_ ($11\bar{2}0$) and Al_2_O_3_ (0001), with two- and three-fold symmetry, respectively, was investigated in situ and in real time by simultaneously monitoring the optical reflection and its anisotropy using DRS and RAS. These parallel measurements allow comprehensive and deep understanding of the details of MoSe_2_ growth. The evolution of the recorded DRS can be well explained by the structure and morphology of MoSe_2_ adlayer at various deposition times. Meanwhile, the RAS signal related to the interface interaction between the orientated MoSe_2_ islands and the Al_2_O_3_ ($11\bar{2}0$) substrate displays highly matched changes with the corresponding DR signal. On the other hand, the comparison of singly oriented monolayer on Al_2_O_3_ ($11\bar{2}0$) and the non-preferential orientation growth on Al_2_O_3_ (0001) surface highlights that Al_2_O_3_ ($11\bar{2}0$) surface is a suitable template for the growth of highly orientated 2D TMDCs. Moreover, this work gives a good example of the complementary method of RAS/DRS in motoring the growth of TMDC materials, which demonstrates its advantage in revealing the growth mechanism and its great potential in controlling the preparation of single-crystalline TMDC monolayers.

## Data Availability

Data are contained within the article and Supplementary Materials.

## Supplementary Materials

The following supporting information can be downloaded at https://www.mdpi.com/article/10.3390/nano14171457/s1, Figure S1: The high-resolution XPS spectra of (a) Mo3d and (b) Se 3d for MoSe_2_ layers on Al_2_O_3_ ($11\bar{2}0$) surface obtained after the deposition time of a–e.
