# Supplementary material for: In Situ Studies on the Influence of Surface Symmetry on the Growth of MoSe2 Monolayer on Sapphire Using Reflectance Anisotropy Spectroscopy and Differential Reflectance Spectroscopy
Source: Nanomaterials (Basel). 2024 Sep 7;14(17):1457. doi: 10.3390/nano14171457 (PMC11397682; doi:10.3390/nano14171457)
Supplement: Supplementary file 1 [file nanomaterials-14-01457-s001.zip › nanomaterials-3163171-supplementary.pdf]

In Situ Studies on the Influence of Surface Symmetry on the Growth of $MoSe_2$ Monolayer on Sapphire Using Reflectance Anisotropy Spectroscopy and Differential Reflectance Spectroscopy

Yufeng Huang [1], Mengjiao Li [2], Zhixin Hu [2], Chunguang Hu [1], Wanfu Shen [1,*], Yanning Li [1,*] and Lidong Sun [3,*]

[1] State Key Laboratory of Precision Measurement Technology and Instruments, School of Precision Instrument and Opto-electronics Engineering, Tianjin University, Tianjin 300072, China
[2] Tianjin Key Laboratory of Low Dimensional Materials Physics and Preparing Technology, Department of Physics, Center for Joint Quantum Studies, Tianjin University, Tianjin 300350, China
[3] Institute of Experimental Physics, Johannes Kepler University Linz, A-4040 Linz, Austria
* Correspondence: wfshen@tju.edu.cn (W.S.); yanningli@tju.edu.cn (Y.L.); lidong.sun@jku.at (L.S.)

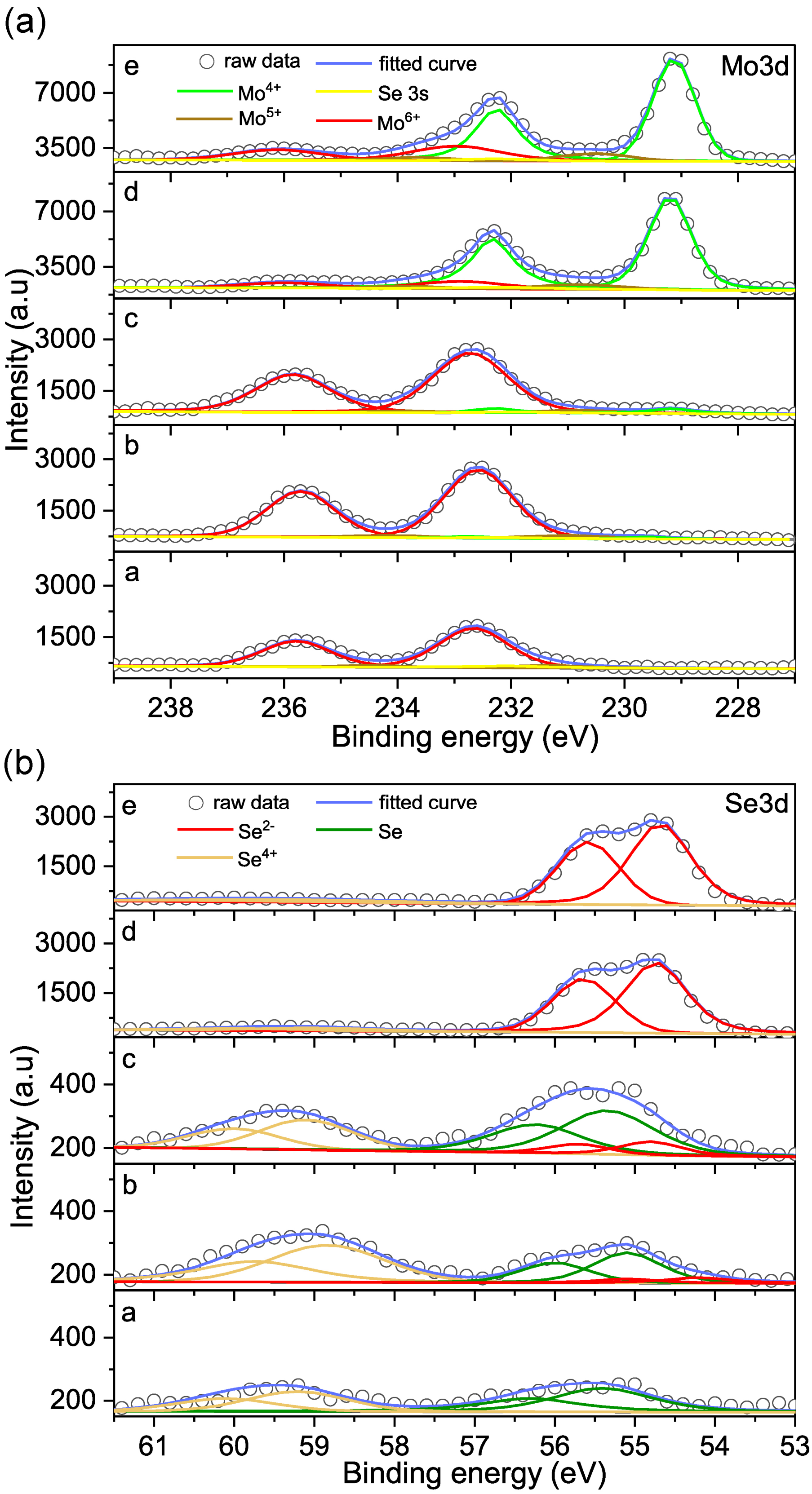

Figure S1. The high-resolution XPS spectra of (**a**) Mo3d and (**b**) Se 3d for $MoSe_2$ layers on Al_2O_3 $(11\bar{2}0)$ surface obtained after the deposition time of a–e.
